# Learning Language, Un/Learning Empathy in Medical School

**DOI:** 10.1007/s11013-023-09830-8

**Published:** 2023-09-19

**Authors:** Seth M. Holmes

**Affiliations:** 1 University of California, Berkeley, 50 University Hall, MC 7360, Berkeley, CA 94720 USA; 2 University of Barcelona, Barcelona, Spain; 3 ICREA Catalan Institution for Research and Advanced Study, Barcelona, Spain

**Keywords:** Empathy, Solidarity, Medical education, Language socialization, Clinical training

## Abstract

This article considers the ways in which empathy for patients and related solidarity with communities may be trained out of medical students during medical school. The article focuses especially on the pre-clinical years of medical school, those that begin with orientation and initiation events such as the White Coat Ceremony. The ethnographic data for the article come from field notes and recordings from my own medical training as well as hundreds of hours of observant participation and interviews with medical students over the past several years. Exploring the framework of language socialization, I argue that learning the verbal, textual and bodily language of medical practice contributes to the increasing experience of separation between physicians and patients. Further considering the ethnographic data, I argue that we also learn a form of empathy limited to performance that short circuits clinical care and the possibility for solidarity for health equity. The article concludes with implications for medical education and the medical social sciences and humanities.

## Introduction: The White Coat Ceremony

It is a sunny August morning in San Francisco before the fog rolls in. There is a cool breeze on the hill between the medical school and hospital on one side and the library and student union food court on the other as new medical students and their guests wait to enter the classroom amphitheater where the White Coat Ceremony will soon take place. My parents, two visiting friends and I take our seats in the steep lecture hall near the back looking down toward the stage over more than one hundred other students and their guests from all over the country.

The Dean of Students^1^ leads us in an updated version of the Hippocratic Oath. Reading aloud, we collectively promise to:

remember that there is an art to medicine as well as a science and that warmth, sympathy and understanding may outweigh the surgeon’s knife or the chemist’s drug.

… not be ashamed to say, ‘I know not,’ nor will I fail to call in my colleague when the skills of another are needed for a patient’s recovery.

…remember that I do not treat a fever chart or a cancerous growth, but a sick human being, whose illness may affect both family and economic stability. My responsibility includes those related problems if I am to care adequately for the sick.

Reading the oath, I feel a sense of awe at the profession I am entering. I am glad this medical school seems to be a progressive institution with leadership focused on health care for all—including stigmatized populations, global health, and those without resources or insurance. I feel a sense of vocation, of calling into this movement to ameliorate sickness and suffering for all and to work in everyday ways in empathy and solidarity with others against inequity and discrimination.

After leading us in the oath, the Dean of Students tells us that we new medical students are “the hub of the wheel” of medicine. He explains that we are the center of the hospital and clinic around which interns, residents, attendings, nurses, patients and families form spokes. The work we do, though at times it might feel like busy-work memorizing facts or sorting through vomitus to find a pill, “is the most important work in the medical center.” He says the hospitals and clinics would not function without us. He calls us to remember this and treat our studies and our conversations and interactions with patients as weighty, fundamental aspects of medicine. We don’t realize yet that “hub of the wheel” will become an oft-repeated phrase among classmates, a meme of our time in medical school. Late at night on overnight shifts or studying in the anatomy lab, we will say it in jest, sarcastically doubting that our memorization or “scut work” matters, while we quietly hope it will all be somehow worth it.

Standing up, wearing our medical student short white coats, we are then asked to read the medical student statement of principles collectively,

…I will not tolerate discrimination on the basis of race, gender, religion, sexual orientation, age, disability, or socioeconomic status.

…I will set patient care as the highest priority….

I will recognize my own limitations and will seek help when my level of experience is inadequate to handle a situation on my own.

…I cannot be compelled to perform procedures or examinations which I feel are unethical or beyond the level of my training.

…I have the right to be challenged to learn, but not abused or humiliated.

Reading the statement of principles, I feel small waves of anxiety. I worry about being abused or humiliated or being compelled to do procedures I am not competent to perform. I wonder why the responsibility to work against each of these seems to be placed on the shoulders of the medical students. I worry about witnessing or even enacting mistreatment and discrimination and think there must be ways for the institutions of medicine, the hospital and the medical school to pledge this same oath. But, quickly, these thoughts and feelings move out of my consciousness as we—new medical students and guests—are congratulated by the Dean of Medical Education. My parents and visiting friends hug me and I say hello to a few classmates I recognize from the orientation events the weekend before. I feel a mix of excitement, pride, and fear—of the unknown, the hints of patient and student abuse, concerns about maintaining empathy and solidarity to counteract discrimination against patients, and questions about my ability to make decisions with life and death implications. I do my best to brush these concerns aside as we walk outside and take pictures in our white coats in the sunny, breezy early afternoon.

The following sections of this article will consider some of the ways in which empathy for patients and related solidarity with communities may be inadvertently trained out of medical students. I focus especially on the pre-clinical years of medical school, those that begin right after orientation and initiation events like the White Coat Ceremony. I analyze the ethnographic data (described below) to understand how future physicians are trained to respond to patients. I engage the framework of language socialization to argue that learning a new biomedical language occurs alongside increasing social distance from patients and communities. I argue that this increasing social distance works alongside an exclusion of important aspects of empathy and the related possibility for solidarity. The article concludes with suggestions for medical education, the medical humanities and social sciences.

## Methods

The ethnographic data come from field notes and recordings from the first years of my own medical training as well as observant participation and interviews with medical students over the past several years. In the classic medical school curriculum model that continues to evolve, the first 12, 18 or 24 months of training are often called “pre-clinical” and focus on medical basic science coursework, including such areas as anatomy, pathophysiology, and pharmacology—often organized around specific organ systems. During these years, medical students are also introduced to clinical practice matters such as patient interviewing, physical examination, charting and patient presentations. During my own medical training—including the medical basic science and practice-oriented courses these first years, I wrote, recorded, and transcribed field notes. I and another MD/PhD anthropologist-in-training recorded 2 h interviews, conversations and reflections with one another every 1 to 2 months during the third and fourth years of medical school (see Holmes & Ponte, 2011). During the final 2 years of clinical rotations as well as the years of internship and residency, I engaged in observant participation (see also Sufrin, 2015) of the medical training of approximately 500 medical students in four medical schools in the USA. During these years, I participated in lectures, panels and small groups and wrote, recorded, and transcribed field notes on medical training. Over the following several years, I have continued to participate in lectures, panels and small groups in over twelve schools of medicine most of which were located in the USA as well as in Latin America and Europe, writing field notes on aspects of the curricula that persist and change over time. During these years, I engaged in observant participation of the education of over 1000 medical trainees. And lastly, over the past 3 years, I recorded 60 to 120 minute ethnographic interviews with 20 medical trainees focused especially on the ways in which the COVID pandemic, the Movement for Black Lives, and changing awareness of racism in medicine related to their experiences of medical training.

Given the focus of this article on subjectivation (see also Holmes et al., 2011) and the experiences through which empathy and possibilities for solidarity relate to the first years of medical training, I rely heavily on the field notes and interviews from my own medical training in which I have a great deal of information on subjective experience. I contextualize and update this data with information from field notes and interviews over the past several years as outlined above in multiple medical schools primarily in the USA and also in Latin America and Europe. In order to protect the identities of the trainees and educators with whom I interacted and whom I observed, I present here ethnographic vignettes as composites of real events.

In the next section, I will consider how empathy is defined by philosophers and social scientists, then some of the ways in which the definition and teaching of empathy in medical education erases core affective and intersubjective aspects. Subsequently, I will explore language socialization in medical education as it occurs alongside increasing social distance between health professional trainees and the patients and communities with whom they work, decreasing the possibility of empathy and solidarity. The article moves on to analyze ethnographic data from clinical evaluations and their focus on performance instead of intersubjective understanding before moving into implications for medical education and beyond.

## Empathy and Medical Training

What is empathy? And why does it matter in medicine? How does it relate to the oath to “remember that there is an art to medicine” … “and that warmth, sympathy and understanding may outweigh the surgeon’s knife or the chemist’s drug”? To the promise to “remember that I do not treat a fever chart or a cancerous growth, but a sick human being, whose illness may affect both family and economic stability”? In many ways, Thokozani Sokela, a Malawian medical student, describing “the heart of the doctor” gives a perfect definition of empathy in the question, “*What if it was me?*” (Wendland, 2010). Empathy involves the ability to imagine what someone else might be experiencing, thinking and feeling (Greater Good Magazine, n.d.). The Merriam-Webster Dictionary indicates “You have empathy for a person when you can imagine how they might feel based on what you know about the person, despite not having those feelings explicitly communicated” (Merriam-Webster, n.d.). The dictionary points out that this is captured by the common phrase “to put yourself in another’s shoes.”

The term, empathy, was introduced into English in the early 1900s to convey the meaning of the German, *Einfühlung* (more literally translated as “in-feeling” or “feeling into”) (Titchener, 1909). Anthropologists and philosophers have debated the meaning of empathy, but generally agree this capacity allows a person to share in another’s subjective experience, without being based purely in common experience (Throop, 2012). Empathy is an imaginative, cognitive, affective, and communicative experience between people who have different experiences or backgrounds. In these ways, we understand empathy to be an incomplete and imperfect (Scarry, 1985; Zahavi, 2003), experientially embodied (Duranti, 2010; Husserl, 1993; Throop, 2012) understanding of what someone else is feeling and *why* (Halpern, 2001a, 2001b). Empathy allows a person to imagine how a certain experience and feeling may impact another’s life priorities and actions. In Throop’s words, “It is…an embodied insight into how feeling [a certain emotion] in a given context may make certain objects, decisions, attitudes, and orientations more or less relevant to [the other person’s] life condition” (2012, p. 410). Empathy is different from projection—one person’s attribution of feelings or thoughts to another without attention or communication about whether or not that matches the other’s experience (Hollan & Throop, 2008), involving instead an ethical stance of openness as well as emotional and intellectual attunement to the other (Kirmayer, 2008; Levinas, 1998).

At the same time, anthropologists point out ways that being understood by others can be harmful in some contexts (Duranti, 1993; Schieffelin & Ochs, 1984; Robbins & Rumsey, 2008; Groark, 2008; Hollan & Throop, 2008). They point out the contextual specificity of empathy, its appropriateness and value or danger (Hollan & Throop, 2008) as well as the ways in which narratives may serve to allow or limit empathy across lines of racialized, classed and gendered difference in clinical practice (Mattingly, 2008). Other social scientists study the situations in which empathy is so completely absent that people engage in collective violence, torture and genocide (Das, 2006; Goleman, 1996; Hinton, 2005; Kleinman et al., 1997; Scheper-Hughes, 1993). Given that empathy allows a degree of understanding of the feelings, thoughts and related priorities and actions of others, it is considered especially important in clinical medicine (Halpern, 2001; Kirmayer, 2008). Given this importance, when and how is empathy present in medical training? And given the possibility of abuse acknowledged in the White Coat Ceremony’s student oath and the potential for violence in the absence of empathy seen in several anthropological studies, when might empathy be excluded or limited in medical training? To explore these questions, we will start by considering the research on empathy in medical education more generally and then move into the ethnographic data.

Several studies indicate worse health outcomes for patients when clinicians *have* less empathy (Mercer & Reynolds, 2002; Hojat, 2018). For example, diabetic patients of physicians who score higher on empathy questionnaires are more likely to have their blood sugar under control (Hojat et al., 2011) and are less likely to need to be hospitalized (Del Canale et al., 2012). Other studies focus on deterioration in quality of care when clinicians *show* less empathy (Hojat et al., 2013) and others indicate that patient health outcomes are worse when the physician is *perceived to be* less empathic (Mercer et al., 2016). And some research suggests that empathy can be increased by education, for example by including medical humanities and medical social science in the curriculum (Batt-Rawden et al., 2013; Riess & Kraft-Todd, 2014). The focus on the *showing of* empathy as opposed to its affective, experiential, intersubjective aspects will be considered further below.

A great deal of research on medical education in the U.S. shows a decrease in empathy between the first and the last year of medical school (e.g. Hojat, 2018). A few studies indicate the decline in empathy occurs most significantly in the third year, when medical students leave classrooms to complete rotations in direct, full-time patient care—precisely when empathy is most important (e.g. Hojat et al., 2009). Other studies show an erosion of empathy starting in the first year of training (Nunes et al., 2011). And some research reveals further declines in empathy during internship and residency (Bellini & Shea, 2005). Research shows higher empathy on average among female trainees and older trainees, though empathy in both groups also declines during training (Nunes et al., 2011). And research indicates that the decline in empathy persists into the future (Bellini & Shea, 2005). Many have studied different aspects of the unofficial, “hidden curriculum” as a way to understand what may lead to this decrease in empathy (e.g. DelVecchio Good, 1995; Hafferty, 1988, 1991, 1998, 2000, 2001a, 2001b) and what Kleinman has poignantly called “moral impoverishment” (2009). In this article, I consider aspects of both the official and the hidden curriculum to understand the decrease in empathy and increased social distance between clinicians and patients and the potential implications for health equity.

At the same time, some research on medical students outside the US shows either no decline in empathy (Quince et al., 2011; Rahimi-Madiseh et al., 2010) or an observable increase in empathy during training (Costa et al., 2013; Kataoka et al., 2009; Magalhães et al., 2011). Some of these studies indicate that empathy declines when quality of life erodes for medical trainees and when burnout develops (Paro et al., 2014). Physician and anthropologist, Claire Wendland, writes of the experiences of medical trainees in Malawi. Unlike their colleagues in the U.S. who experience a decline in empathy and an alienation between physician and patient, the Malawian medical students who learn biomedical diagnoses and treatments from many of the same books used in the U.S. develop what they characterize as “heart” or “love” for patients and society. One of the medical students she interviews (quoted above) explains that the heart of the physician is “What if it was me?” (Wendland, 2010, p. 204). These medical students understand that they are learning not only how to diagnose and treat disease in the individual body but also how to “see deeply into the pathologies of society” (*Ibid.*, 204). They understand and diagnose the cause of sickness and suffering “squarely in national and global structures”, and this “aligns doctors and patients together in solidarity against the system of global exploitation and inequity—or national failure and corruption” (*Ibid,* 205).

Wendland argues that we must consider (1) the ways in which stories told about medicine in different contexts provide meaning to trainees as well as (2) the material circumstances of medical training. These aspects may help us understand how biomedical practitioners learning from the same books and principles of diagnosis and treatment have such different subjectivations, habituses, and gazes in different contexts. Wendland writes of stories of doctors in Africa who fought for health, food, housing and education for all. These stories provide meaning in the midst of demanding and difficult years of training. She writes of the challenging material circumstances in which Malawian medical students work, often without the medicines or surgical implements necessary to perform the treatment they spent hours and hours memorizing. They work in underfunded hospital wards where patients’ families must provide sheets, food and a great deal of nursing care. And they come away from this narrative and material experience with a sense of both *empathy* and *solidarity* with patients to improve society and the health system.

How do the existing forms of language, words and phrases that condition narratives in medicine in the U.S. shape the meaning we make of our work as medical trainees? These aspects will be the focus of the next section. The material circumstances of training—including current forms of capitalism, the individualism it promotes, the racism and discrimination with which it has been bound, as well as private insurance systems and the proliferation of technology in the U.S.—will be considered further in future publications from this ongoing research.

## Learning the Language of Medicine

The first year of medical school, I have class from 8AM to 5PM with 1 h for lunch on Mondays, Tuesdays, Thursdays and Fridays. On Wednesdays, I have class from 8AM to noon. Most of the classes are large lectures in an auditorium with my class of roughly 150 medical students. These include disciplines like Physiology, Pharmacology, and Embryology. Each of these classes requires an incredible amount of homework and studying that I write on a calendar on my wall. Some of the classes are “problem-based learning” small group discussions of clinical cases or sometimes more general discussions related to becoming a doctor. We have small groups where we practice clinical skills—for example, listening to hearts with stethoscopes, looking into eyes with ophthalmoscopes, checking blood pressures with sphygmomanometers—usually on each other. We have several hours of anatomy lab each week, dissecting cadavers in groups of four students—after which we return for several more hours to study and memorize. Over the first 2 years, our courses move slowly from more basic science with intermixed cases toward more practical clinical knowledge with intermixed reminders of the basic science underlying the clinical diseases and treatments. Increasingly, we learn to and practice interacting with patients, “interviewing” and verbally “presenting” them to our medical superiors—interns, residents, and attending physicians.

In lectures and small groups, the instructors—mostly medical doctors themselves—routinely use words that most of us have never heard. The instructors use these words without seeming to remember in those moments that other people, including new medical students, might not know these specialized medical terms. And for some reason, I am one of the students who is least embarrassed to admit when I do not know a word (or perhaps least familiar with medical vocabulary). After class, several students tell me they did not know the terms either and were relieved that “someone else asks the stupid questions.”

In lectures, textbooks, and clinical skills small groups, we learn entirely new words for things most of us did not previously know—surgical procedures such as “episiotomy” or anatomical names like “Virchow’s triad”. We also learn new words for entities we already knew: for example, “erythematous” instead of red; “indurated” instead of hardened; “edema” instead of swelling; “xerosis” instead of dry skin; “cerumen” instead of ear wax; “epistaxis” instead of nosebleed; “fasciculation” instead of muscle twitch; “rhinorrhea” instead of runny nose; “emesis” instead of vomiting. The new words for things we used to describe in regular English seem unending and often unnecessary. At the same time, we learn that some words we use in everyday English mean something entirely different in medicine. For example, “sensitive” and “specific” take on new, strict meanings based on statistical equations to indicate how precise a medical test is in a detailed, particular aspect. These definitions are so new, that most of us memorize mnemonics to remember them. “SNOUT” helps me remember that “sensitivity” helps rule “out” disease and “SPIN” that “specificity” helps rule it “in”.

Even though in elementary school grammar I was taught repeatedly to avoid double negatives and to find more precise phrases to describe what I meant, I now learn that many double negatives are commonplace and even expected in medical communication. For example, “not unlikely” and “not unexpected” are appropriate, ubiquitous ways to communicate that a certain outcome is somewhere between *unlikely* and *likely* or between *unexpected* and *expected*. Contradicting our previous grammar lessons, we find that in the language of medical prognosis—whose temporality is imprecise and suspended in probabilities, double negatives are not only tolerable but standard.

And as we proceed in medical training, we learn new terms for important diseases and syndromes that sound like words we already knew. For example, “TACO” now means “transfusion-associated circulatory overload”. We learn that it can be critical to differentiate this potentially life-threatening syndrome from “TRALI” (pronounced “trolley”) or “transfusion-related acute lung injury”. During lectures, small groups and clinical rotations, our ability to differentiate between TACO and TRALI are frequently tested publicly in what I later learn are colloquially known as “pimping” sessions on clinical rotations and in “Morbidity and Mortality” noon conferences over lunch. As we will explore further below, the process of learning a specialized language involves becoming a member of the medical community and may play into subtle forms of separation from patients and their families.

## Language Socialization

*Language socialization* refers to the simultaneous, reciprocal process of learning to use a language in a given society and becoming a member of that society through the use of language. As explained by leaders in the field, “the process of becoming a competent member of society is realized to a large extent through language, by acquiring knowledge of its functions, social distribution, and interpretations in and across socially defined situations, i.e., through exchanges of language in particular social situations” (Ochs & Schieffelin, 1984). Research has shown the ways in which children are socialized into their roles as patients through language (Stivers, 2011) and the ways in which time management and treatment disagreements are managed linguistically by doctors and patients (Sterponi et al., 2019, 2021). As opposed to the classic focus on “language acquisition” as a solely technical endeavor of learning terms and grammar, the study of language socialization highlights how “the process of acquiring language is deeply affected by the process of becoming a competent member of society” (Ochs & Schieffelin, 1984) and vice versa.

This is clearly the case with medical language. One could use vernacular words like “red” and “swollen” to communicate the appearance of a rash. However, medical students learn through readings, lectures, small group discussions, and apprenticeship in clinical situations to translate this into a medical register. Learning this medical language is one means by which we also become—and become recognized as—members of the medical community. Correctly utilizing this language and its communicative practices signals expertise and belonging. Throughout my first year of medical school, it becomes clear to me that the purpose of using words like “erythematous” and “edematous” is not just to describe the appearance of a body part (that could have been described with more familiar words), but importantly to be recognized as someone who belongs in medicine, someone who should pass this year of medical school and be allowed to continue training. As medical training continues, it then becomes more important to show my correct employment and differentiation between “sensitive” versus “specific” with regard to medical tests and then, especially in internship and residency, the different causes, appearances and treatments of “TRALI” versus “TACO”.

While language socialization scholars indicate that learning a language is part and parcel of becoming a member of a society, they also show how learning a language changes our perceptions and even ourselves. For example, students of Traditional Chinese Medicine in the U.S. experience their own transformation as people and healers as they practice translating Chinese diagnostic terms into English (Pritzker, 2014). And children in California learn which of them and their parents belong (and which do not) in specific places and countries in the process of learning languages (Baquedano-López & Janetti, 2017). This highlights the simultaneity of learning and experiencing hierarchy, exclusion and discrimination—including nationalism and racism—in the process of language socialization.

As another example, it becomes increasingly difficult to think of or be upset by “genocide”, “murder”, or “war” as one learns the language of defense intellectuals and becomes skilled in communicating about “counterforce exchanges”, “minimum deterrent postures”, “clean bombs” such as “The Peacekeeper”, “surgically clean strikes”, and “collateral damage” (Cohn, 1987, pp. 687–718). As Cohn writes,

There are no ways to describe the phenomena represented in the first with the language of the second. Learning to speak the language of defense analysts is not a conscious, cold-blooded decision to ignore the effects of nuclear weapons on real live human beings, to ignore the sensory, the emotional experience, the human impact. It is simply learning a new language, but by the time you are through, the content of what you can talk about is monumentally different, as is the perspective from which you speak (Cohn, 1987, p. 705).

Taken together, this scholarship indicates some of the important ways we may change as we learn languages. We become—and become recognized as—members of a group. At the same time, we ourselves are transformed when we learn new languages; we learn lessons of hierarchy and the limits of inclusion; and our perceptions may be restructured with potentially critical implications. In medical training, of course learning the language of medicine is part of how we become recognized as future physicians who should be allowed to graduate to the next year of medical school. But, what else changes in medical students, our perceptions of patients and the social world, and our understandings of hierarchy, inequity and the possibilities or difficulties of empathy and solidarity as we learn medical language?

On the most basic level, we learn that we are experts on health and bodies. Of course, in many ways, this is true and this is one primary goal of our training and one of the things patients often expect of us. In explicit and implicit ways, we also learn that patients and their families do not understand their own health and bodies. Here, I am not implying that patients should be understood to have the same knowledge or expertise as health professionals. Rather, I am focusing on the ways in which expertise—in language specifically—leads to different layers of separation between one group and another. On one level, there is a symbolic level of separation in which medical professionals (trainees and physicians) are treated as the primary subjects. In interviews and conversations recorded with medical students, residents and interns, almost all of the subjects of verbs were medical professionals. It was very rare for a patient to be the subject of a verb in the statements by medical students, interns, residents or attendings. The vast majority of the decision-making and action was attributed to medical professionals only. In these interviews and conversations, the relationships within the medical team and between medical teams were also treated as by far the most important (see also Konner, 1988), which were sometimes a source of pride or contentment and other times a source of great anxiety. The statements by and dialogues between medical professionals indicate what has been shown in the literature, that we begin to identify increasingly with physicians and less and less with the patients with whom we work (e.g. Ibid.). While gaining expertise in medicine is extremely important, I argue that forms of our learning involve increasing separation from patients on multiple levels that may decrease empathy and solidarity and may, ironically, worsen the care we attempt to provide. In a later section, I will also argue that language socialization is an important part of the development of what medical social scientists often call the clinical or medical gaze.

As with other forms of expertise that involve a “retreat from standard English” (Nader, 2018), the specialized language that may be necessary for communicating critical details can also serve to exclude lay people from important conversations. The exclusion enacted by expert medical jargon can reinforce power hierarchies in clinical interactions, making it more difficult for patients and families to understand the possibilities and plans for diagnosis and treatment and therefore incredibly challenging to engage in “shared decision-making”—an important model for ethical interactions between health professionals and patients. Yet, it is impossible for decision-making to be truly shared when one group is excluded from vital, basic information in the conversation. This exclusion from information exchange forms a more practical level of increasing separation between medical trainees and patients and their families.

Perhaps the most troubling aspect of this medico-linguistic exclusion of patients and families is that we tend to become so used to medical jargon that we often do not notice we are using it. Medical terms become so commonplace that we forget others may not know them. I remember multiple times during medical school and increasingly during internship and residency explaining something to a patient or family, only to realize after the fact that I had used terms most people do not know. For example, in medicine, we often discuss the “differential diagnosis”, meaning all the possible diagnoses that could be causing a given health problem or symptom. We commonly refer to this as the “differential” and we discuss the diseases “on the differential.” More than once, I tried to be helpful by explaining the important diagnostic causes “on the differential” to a patient or family without being aware that “differential” is not clear in standard English. Sometimes patients or family members pointed out my jargon or asked questions. Most often, they did not—and only sometimes later did I realize the words I used may have fostered misunderstandings and exclusion.

This exclusion became most clear to me during my internal medicine residency, when my boyfriend at the time was put on a ventilator after being exposed to a rare lung virus while traveling, making it impossible for his lungs to breathe on their own. He was in great shape—the picture of health and athleticism—and had been on a long-distance multi-week bicycle ride when he (I will call him “Lance”) became sick. While the Intensive Care Unit medical team ran tests and worked extremely hard to care for Lance, his parents tried to make sense of the sudden serious condition of their son who had previously been so healthy. After seeing my own patients in the hospital during the day, I went to visit Lance and his parents in the ICU at the end of the day.

Even though the ICU attending physician was impressively knowledgeable and caring and spent time each morning updating the loved ones and families of the patients, Lance’s parents routinely left these meetings very confused. Each evening, I spent time going back and forth between the parents in the waiting room and the ICU team to pass on questions and responses—to translate. Lance’s lungs had stopped working on their own and he was put on a ventilator (breathing machine). No test could find a cause; no bacteria or viruses were detected despite an exhaustive search. Because of the spread-out appearance of the pneumonia fluid in the lungs on the imaging scans, the ICU team considered the cause to be most likely a virus. In meetings with the family, the ICU team explained to the parents that he had an ”idiopathic viral pneumonia” and later said that his pneumonia was “viral NOS” (pronounced “viral N, O, S”). They went on to state that Lance had a “precarious prognosis”. While I was used to each of these terms, the family did not know that “idiopathic” means “we do not know the cause”, nor that “NOS” means “Not Otherwise Specified”, essentially saying again that “we do not know which virus is the cause.” And “precarious prognosis” indicated that Lance’s condition was so extremely severe that the team did not yet know if he would recover or not. I found myself explaining each of these important, uncomfortable meanings to Lance’s parents. (Thankfully, he recovered back to his full bicyclist health in a matter of weeks.) In the process, it became even more clear to me that the ways we physicians are trained to communicate often do not make sense in standard English.

According to medical sociologists, the specialized language we learn also allows us to balance detachment and care in order to navigate potentially emotionally taxing experiences day after day while working with patients and their loved ones (Fox, 1959; Leif & Fox, 1963). Fox calls this attitude “detached concern” (Fox, 1959) and indicates that it is a “dynamic balance” in the emotional and practical experience of medical training (see also Cadge & Hammonds, 2012). Other medical humanities scholars argue that medical training has shifted too far toward detachment and that developing the capacity for empathy among medical trainees will correct this balance and improve relationships between medical professionals and patients (Halpern, 2011). The process of language socialization that contributes to the symbolic, experiential and communicative separation of medical trainees from patients and their families as discussed above may be part of this shift further toward detachment and away from empathy. Next, we consider some of the explicit ways empathy has been taught in medical education before discussing other aspects of the explicit and hidden curriculum that may foster the empathy decline.

## Learning to Show Empathy

Midway through our first year of medical school, we receive a lecture and small group training on empathy. The guest lecturer, a friendly white middle-aged psychiatrist affiliated with the School of Medicine, shows us PowerPoint slides documenting the decline in empathy during medical school. He shows slides about patient dissatisfaction with medical care: patients feeling like they are being treated like machines to be fixed instead of human beings to be cared for and patients feeling like they are being rushed through the appointment without being listened to fully by the doctor. He shows research indicating that patients often leave clinic appointments with the disconcerting feeling that the physician is paying more attention to the computer screen than to the person in front of them. Without considering other aspects of the context (lack of time in market-driven healthcare, increasing health information requirements, etc.), the lecturer explains that these negative experiences are due to a lack of empathy shown by physicians. To counteract these alienating experiences of patients, he tells us he will train us to *show* empathy.

The expert lecturer standing in front of the room then shares one of the acronyms to train health professional students to show empathy. As he explains that we should memorize the acronym to show empathy, the word “NURSE” appears in large, capital letters in a vertical column on the left of the screen. As he explains the meaning behind each letter, the following words appear: “Naming”; “Understanding”; “Respect”; “Supporting”; “Exploring”. Like most acronyms used to teach empathy to health professional students, this framework focuses on what to say, what to communicate with words to show empathy. The first step is to “Name” what the patient said or asked, including any emotions they shared. Some of the suggested phrases include “You are asking for the results?”; “It sounds like you are angry/frustrated.”; “You seem very sad.” The next step is to show “Understanding” by “validating the emotion” the patient shared. Suggested phrases are “This helps me understand what you are thinking.”; “I know this must be really hard for you but is there anyone I should contact for you?”; “I can’t imagine how hard this must be for you.” The third step is to show “Respect” by “prais[ing] patient for coping” with phrases such as “I think you have done a great job with this” or “I can see you have really been trying to follow our instructions.” The fourth step is to “show/provide Support” with phrases such as “I will do my best to make sure you have what you need.” And the final step for us to memorize is “Exploring” the patient’s emotions by asking “open-ended questions” such as “Could you say more about what you mean when you say that…” or “Tell me more about what you are afraid of” (Hannan et al., 2019). The guest lecturer explains that if we practice these suggested phrases, we will be showing empathy to our patients and they will experience better medical care.

During the lecture, it is striking to me that virtually nothing is said about emotions, understanding or thoughts on the part of the health professional. Even though this affective aspect is considered core to empathy by social scientists and philosophers, the focus in medical training is almost exclusively on practices: statements, questions, keywords, and suggested phrases. While there were many other large and small-group discussions with patients about health, disease and health care that helped us imagine their experiences, I wonder why the lecture designed explicitly to teach empathy did not involve us hearing from patients about their experiences of illness and treatment. I imagine the session on empathy involving a lecture and hearing from patients, which might help us ask “What if it was me?” and “put ourselves in another’s shoes.” I wonder how it would feel if the lecturer asked us to talk about our own or our family members’ or friends’ experiences of illness and health care. And I wonder how we medical students would receive it if, after hearing from patients and/or sharing stories from our own lives or communities, the facilitator were to encourage us to remember what patients might feel, how those feelings might affect their priorities and lives and to respond to that understanding. I imagine this could help us develop empathy and practice the oath we took at the beginning of medical school to “remember…that warmth, sympathy and understanding may outweigh the surgeon’s knife or the chemist’s drug.”

Another acronym commonly used to teach medical students is “EMPATHY” with each letter standing for “Eye contact”; “Muscles of facial expression”; “Posture”; “Affect”; “Tone”; “Hearing the whole patient”; “Your response.” This tool is intended “to enhance nonverbal communication between clinicians and their patients” (Riess & Kraft-Todd, 2014). While “Hearing the whole patient” gestures toward broader forms of communication that could include the “understanding” generally considered part of empathy, the rest of the mnemonic asks clinicians to memorize not just statements and questions but also how to stand, what tone of voice to use, and which muscles to activate in order to be *perceived as* empathetic.

The lecturer tells us that research shows that patients respond well to the suggested areas of communication he just taught us. However, after class, the reduction of the philosophical concept and social, interpersonal reality of empathy to memorized practices leaves many of my classmates uneasy. The rest of the week, my classmates and I joke about how awkward it was to learn an acronym to memorize in order to be more caring human beings. Some of my classmates argue that empathy cannot be memorized and that maybe the acronym is good because the people who are already empathetic will not need it, but the people who are not empathetic can at least learn a few words or phrases to use. Other classmates and friends discuss the lecture slides about the decline in empathy during medical school and wonder about the factors contributing to this issue. Most classmates say that the empathy acronym will help our scores on the Clinical Practical Exam, so we can “pick up a few extra points” showing “fake empathy” (Jeffrey, 2017).

We are taught, not so subtly, to act and speak in ways that convey empathy more than we are encouraged to understand or feel in ways considered by social theorists to be inherent to empathy itself. This focus on showing empathy instead of on understanding patients’ experiences and lives and feeling in response to that understanding may help foster our increasing separation from patients on multiple levels. Next, we will consider the Clinical Practical Exam before discussing further the implications of the focus in medical education on showing as opposed to feeling empathy.

## The Clinical Practical Exam

One morning part-way through my clinical rotations, I arrive at the medical school dressed in a button-down shirt, slacks, dress shoes, and white coat with my stethoscope around my neck. I will be tested today on my ability to act like a medical student; I do my best to look the part. We are going to have the Clinical Practical Exam—that we all call the “CPX”—during which we see eight actors, each paid to portray a specific patient case. Such actors are called “standardized patients” in medical education. We will be video recorded with 15 minutes to meet each actor-patient, interview them about their symptoms and medical history, do a physical exam, and explain to them a diagnosis and treatment plan. After each actor-patient, we will have 10 minutes at a computer to answer questions about the patient, their condition, and our plan. This type of “Observed Structured Clinical Examination” has been adopted increasingly in health professions education around the world over the past few decades as a statistically reliable way to test the clinical competence of students (see Hodges & McIlroy, 2003; Wani, 2015).

After spending over 5 years completing my PhD in anthropology—with a few “preceptorships” during which I followed a physician for one half-day each week in tuberculosis clinic, jail clinic, and homeless clinic—I feel nervous about my inevitably rusty clinical skills. Whereas my medical school classmates completed 12 months of clinical rotations and had a practice mini-CPX, I recently returned from graduate school in anthropology and was not in medical school yet at the time of the practice mini-CPX. Before the CPX, I emailed two of the clinical skills professors and asked how I should prepare. They suggested I read Bates’ *Guide to Physical Examination* (Bickley et al., 2021), which is a 1,064 page textbook full of detailed information on every imaginable clinical exam skill. Amid my full-time clinical rotation duties and preparing for the feared specialty-focused “shelf exams” at the end of each rotation, I studied as much as I could of Bates. In the back of my mind as I prepared for the CPX, I also remembered a medical student friend in tears after she received feedback from the standardized patients.

As we wait for the CPX to begin, I see one student outside each exam room near a computer on a cart—affectionately called a “computer on wheels” or “COW”. We are instructed to wait to look at the “Chief Complaint” of the patient until the timer is started, and then we will be permitted to enter the room to meet the standardized patient. My imposter syndrome is activated as I wait to look at the basic information about the first standardized patient I will see. Once we are allowed to look at the first patient’s “Chief Complaint” or the reason they came to clinic, I am pleasantly surprised by how much I remember. The first standardized patient I will see is a 35-year-old man with back pain. As I think quickly about some of the conditions that could cause this, what questions I should ask and which parts of the physical exam I should perform, I see that most of my classmates have already entered their exam rooms. I take quick notes on my pad of paper, enter the room and introduce myself as Student Doctor Holmes.

After asking questions about the patient’s pain, his medical history and his work, I conduct an exam of his back and tell him my plan for an X-ray to help determine the diagnosis and treatment of his pain and preventive measures to avoid future back problems. I see seven more standardized patients with abdominal pain, chest pain (concerning for a heart attack), bloody cough (concerning for tuberculosis), depression, a family history of breast cancer, and diabetes. As I see each standardized patient, I am pleased with how much I remember: potential common causes, potential serious causes, physical exam skills, and next steps for diagnostic tests or treatment.

After responding on the computer to questions about the first patient and hearing the buzzer go off, I enter the second exam room and introduce myself as I begin to wash my hands. I do not want to wash my hands after shaking hands with the patient—which I imagine would imply that I am worried about them being dirty—and I want them to see me wash my hands before I examine them. At the same time, I want to introduce myself right away to avoid being rude—and to avoid wasting any of the precious 15 min. However, the standardized patient interrupts me and tells me it is rude to talk with her while washing my hands. She tells me I am unprofessional and I should wait. I am confused about what I should take away from this interaction; it does not seem ideal to wait to introduce myself or to wait to wash my hands. After the computer questions and the buzzer, the next standardized patient cries and I do my best to comfort her while also keeping the interaction moving forward within the 15-min limit we have. I quickly wonder what it is like to cry over and over with each medical student who comes in the exam room. I push this thought aside to avoid falling too far behind.

Each standardized patient case is designed to test our patient interview skills, physical exam skills, diagnostic and treatment knowledge, and some specific aspects of what is often called “physicianship” in medical school. Physicianship, also referred to as “professionalism”, is often considered a “core competency” for medical students that is “fundamental to the practice of medicine” according to the medical schools where I have worked. Serious or recurrent problems with physicianship are grounds for probation or dismissal from most medical schools. In the medical schools where I have worked, the official written examples of unprofessional behaviors that could lead to a “Physicianship Evaluation Form” include such things as “repeated tardiness”, “unexcused absence from a required activity”, “disruptive behavior”, “academic dishonesty (cheating or plagiarism)”, “abdication of responsibility for patients”, “harassment or violence”. However, there are more subtle or perhaps personal, interpersonal and affective aspects that form part of physicianship. During our second year of medical school, we are given the case of a medical student to read in order to learn how to avoid poor physicianship. The student in the case is part of a medical team taking care of a patient with cancer. The case describes the medical student as identifying “too closely” with the patient and “not identifying enough” with the rest of the medical team. This interpersonal aspect of physicianship seems to reflect the school’s expectation for us to develop separation from patients as we join the medical community, perhaps showing increasing detachment and avoiding too much empathy or solidarity.

After my experience rushing through the CPX of eight standardized patients in a row, I feel proud of how much I knew and what I was able to do with only 15 minutes in each exam room. The following week, I receive my grade that was 9% below the class average—which seems pretty good to me, considering I just spent 5 years doing anthropology. However, I am then informed that I failed the CPX. The grade has no other information about what was expected, what I had missed, or what I should study to improve. I receive an email that I will not be given any further information about what was expected or how to improve for several months. I am told that after I find out what I missed, I will have to wait another several months before I will be required to re-take the CPX.

The only comments available to me from the CPX are from the standardized patients. One standardized patient indicated that they felt “connected by warm manner, soft voice and eye-contact” and another responded “I felt student listen to me and allowed me to share my feelings. I would come back to see this physician.” However, another standardized patient indicated they were “concerned because student was very soft spoken and seemed reserved or nervous, not very authoritative seeming.” Another standardized patient indicated that I used the words “I think” and my “body language seemed unsure: example—leaned on side of feet while explaining diagnosis.” Another patient indicated that I did not explain the term, “endorphins”. While this last comment points out how medical jargon can exclude patients, the other comments show expectations of very specific ways of acting, holding one’s body or *showing* emotions and competence. They indicate the expectations of *performing* confidence and knowledge even when one does not feel it, seemingly contradicting the oath we took that “I will not be ashamed to say, ‘I know not’.” Instead, we are not-so-subtly trained to act like we know it all. Reading the comments and knowing I failed this test, my confidence is shot. Yet, I prepare to move immediately on to my 8-week intense hospital surgery rotation.

While medical education scholars tend to argue that these Observed Structured Clinical Exams are statistically reliable ways to test clinical competence as mentioned above, some of the literature also shows a great deal of anxiety caused by the exam—especially without practice exams ahead of time (see Khan et al., 2016). However, I do not mean to argue here about the validity of testing competence nor of anxiety per se (though that was certainly part of the experience). Rather, I am interested in the subtle ways in which the CPX—along with professionalism and other aspects of medical training—encourages a move toward how we *perform*, *act*, and *show* and away from how we understand or feel. The focus on how we show competence even includes our competence at *showing* empathy. However, we are subtly encouraged away from understanding our patients as whole people including how their emotions and experiences might affect their priorities and orientations toward their health and future. We are taught in subtle, repeated ways that the affective, intersubjective aspects of empathy core to definitions by social scientists and philosophers are not part of our role and may even cause us problems in the area of professionalism. This general focus—in the CPX, in empathy lectures and acronyms, and in professionalism education—on *showing* instead of *feeling* may foster separation from patients at levels of identity and affect while language socialization enacts separation at symbolic and practical communicative levels.

## Language Socialization and Medical Culture

The framework of language socialization described earlier is not limited to learning specific new terms or phrases, but also includes learning broader ways of constructing and telling stories, inflecting surety or doubt, signaling deference or defiance. As described earlier, language socialization scholars indicate that these broader ways of learning to organize and construct sentences and narratives shift our perceptions of ourselves, others and the social world.

I consider language socialization—the sociolinguistic process incorporating us into the medical community while teaching us who is the subject and implicitly, the object, of medical discourse—to be one integral aspect by which the clinical gaze is produced. Social scientists have utilized the concept of the clinical or medical gaze to analyze the ways in which medicine tends to individualize, objectify, reduce and depoliticize health (e.g. Davenport, 2000; Holmes, 2012, 2013). In Foucault’s description, the primary clinical question changed from “what is wrong?” to “where does it hurt?” (1973, p. vi), seeing and treating the patient less as a human being in social context and more as a series of objects making up a body. Foucault shows ways in which this change was necessary for the birth of positivist science and contemporary forms of medical knowledge while it also produces reductive understandings of health, the body, and the human. The clinical gaze, for Foucualt, is not just literal seeing, but rather a whole host of cognitive, physical, logistical, technical, discursive practices that organize the execution of medical care and the relationship between clinician and patient. Language socialization is an especially important social, cognitive, technical, and discursive process through which the clinical gaze is produced. Paying attention to this sociolinguistic process and its implications, we will be able to more fully understand the clinical gaze and how it comes to be embodied and enacted by clinicians on individual and collective levels.

On one hand, in medical school we learn repeatedly that human beings are primarily individuals with bodies and health that are to be examined, understood, treated and healed individually. Patient cases we discuss in small groups are almost always focused exclusively on the individual and their body parts or physiology. We recognize as a general rule that any implications of the injury or sickness or of the treatment on the family or community should be left out. Despite the oath we took aloud in the White Coat Ceremony to “remember that I…treat…a sick human being, whose illness may affect both family and economic stability” and that “my responsibility includes those related problems if I am to care adequately for the sick,” the connections between patients, sickness, economics and family are repeatedly erased from consideration as we move through medical school. The medical social sciences could remind us that these implications are extremely important for one’s health and well-being: from the inability to pay rent while one is in the hospital and unable to work to the impossibility of affording both expensive treatments and enough food for a family to eat. There may even be ways in which social, political and economic issues caused or exacerbated the sickness. However, we learn that the primary focus of any discussion of a patient’s sickness or injury should not consider implications beyond the individual body part. Even in the “social history” we are instructed to include when we “intake” a new patient, we are trained not to include social factors, but rather to focus almost exclusively on limited “health-related behaviors”.

Roughly twice a year during my first 2 years of medical school and then during 2 week “intersessions” halfway through third and fourth year hospital rotations, we have small group discussions of patient cases written intentionally to bring up ethical quandaries related to the social and economic implications of sickness. While I find these case discussions especially interesting and helpful, their stark divergence from the ubiquitous cases we routinely discuss reinforces my understanding that these are only small exceptions to the broad rule of focusing on individual bodies and health. As shown by scholars around the world, many other healing systems include detailed and explicit consideration of the social world in the practice of diagnosis and intervention (Kalofonos, 2021). However, two of the “tenacious assumptions of western medicine” are that the individual is more important than and prior to society and that nature is autonomous from and prior to society (Gordon, 1988). As we learn medical ways to communicate about patients as cases, we learn what to focus on and what to leave out (Holmes & Ponte, 2011). We are subtly and explicitly trained not to bring in discussions of social issues as we learn to focus on the individual and their body. In these ways, we are socialized into individualism and “naturalism” (Gordon, 1988). Given that the social and economic implications of health, disease and health care are part of what patients and families experience, the repeated subtle ways medical students are encouraged not to consider or discuss these aspects fosters further separation from patients.

Following the propensity of medicine to break complex realities into separate elements and study quantifiable variables (c.f. Holmes & Ponte, 2011), most medical education researchers define empathy with the following separated, specific components that will be discussed further below: (1) the ability to *understand* the patient’s perspectives, concerns and feelings, (2) the capacity to *communicate* that understanding in order to check its accuracy, and (3) the *intention* and capability to act on that understanding in a helpful way that prevents or alleviates pain and suffering for the patient. Some call these the *cognitive, behavioral* and *moral* components of empathy. Interestingly as discussed above, the *emotional*, *affective* (including the phenomenologically intersubjective embodied) component commonly understood as core to empathy, the ability to experience and share in another’s feelings and experiences, is most commonly left unconsidered in medical practice (cf. Hojat et al., 2009; Mercer & Reynolds, 2002; Neumann et al., 2012). On multiple levels, medical education scholarship and practice tends to reduce empathy to a technical and measurable, performable skill, not a multi-faceted capacity to develop within oneself. This reduction of empathy to performance and the multi-layered separation from patients through language socialization become part of the clinical gaze through which we perceive and respond to patients.

## Implications: Witnessing, the Clinical Gaze and Social Medicine

While the learning of words, phrases, and the format of cases teaches us individualism and naturalism and excludes patients and families from effective shared-decision making, this process also limits the relationality and affective resonances of empathy. As we learn the language of medicine, we are taught empathy as a performance of suggested phrases and muscle activations. Our lecture explicitly on empathy and the acronyms meant to teach it focus on *showing* while our Clinical Practical Exam reinforces the focus on what we show and perform as opposed to how well we understand the patient and their experience. The lost aspect of empathy in medical education is precisely that relational, interpersonal “putting oneself in another’s shoes” and asking “What if that was me?” And our professionalism training explicitly warns us of the dangers of not separating enough from patients.

Together, these experiences during medical training—language socialization with its symbolic and practical separation from patients, definitions of empathy focused almost exclusively on performance, reinforcement of the focus on performance in important clinical evaluations, repeated exclusion of social context from cases, and encouragement of identity detachment from patients in professionalism training—foster separation from patients on multiple levels. We are subjectivated into a clinician self (see also Holmes et al., 2011) who is separated symbolically and communicatively and detached affectively from patients and understands empathy as primarily a performance. And the performance-oriented version of empathy in medical education pedagogy and scholarship seems to be impotent to counteract experiences of burnout and moral injury (Watson et al., 2020). Without its core intersubjective, affective aspect, empathy is an empty shell that likely works against solidarity with patients and families that could have led to health systems, social and/or political change toward health equity.

In the midst of the ways in which medical training led me toward further separation from patients and toward perceiving patients primarily as individuals or bodies with decreasing awareness of their family and social context, I am grateful for experiences of electives and mentors who modeled different ways of practicing medical care. Some of these physicians seem to “remember that there is an art to medicine as well as a science, and that warmth, sympathy and understanding may outweigh the surgeon’s knife or chemist’s drug.” They also model treating “a sick human being” and “not…a fever chart or a cancerous growth.” These mentors remind me of Davenport’s description of a clinical training site where the attending physicians and medical students try to practice “witnessing” of the whole person and the social conditions that exacerbate their sickness (2000). She writes how, even in this clinical site actively and intentionally working toward a more socially aware and holistic form of care, the practice of medicine—including medical language—also fosters the medical gaze that simultaneously individualizes and objectifies patients. Throughout my training, I felt this push and pull between the desire to treat each patient like a person, an equal within a social context that matters, and the need to learn the language and practices of medicine that may also involve individualizing and objectifying in order to be permitted to become a physician. These mentors and the training site described by Davenport offer different narratives—and even sometimes different material and institutional realities; the two aspects Wendland argues could explain the increase in empathy among the medical students with whom she worked.

So, how could medical education counter this increasing affective, symbolic and communicative separation from patients and its correlated decline in empathy? Scholars in the medical humanities have explored many opportunities for the development of a fuller form of empathy in trainees (e.g. Cao & Chen, 2021; Garden, 2009; Halpern, 2001b) with some evidence of success (e.g. Graham et al., 2016). Unfortunately, such medical humanities curricular innovations are often left at the margins of the curriculum signaling to students that the forms of understanding and empathy they teach are not of core importance. It will be important for leaders in medical education to seek ways to incorporate knowledge and methods from the medical humanities more directly into the core curricular experience. At the same time, medical social scientists have argued for a focus not so much on shared humanity between health professionals and patients—due to both critiques of the ways in which racialization, nationalism and capitalism exclude certain people from the category “human” and increasing awareness of the ecological connections in this more than human world, but instead on the impact of and resistance to the forces that cause inequity, disease and injury in the first place (Dubal, 2018). They argue that an awareness of the social allows for more full understanding of patients and solidarity with patients for important social, health and health care change (Dubal, 2018, “Conclusion”). This awareness of the importance of the social could counteract the reductive tendencies of the clinical gaze as well as the separation fostered by the language socialization that helps produce that gaze in the first place (see also Stonington, 2020, “Conclusion”). On a most basic level, the “social history” could include social factors that the patient and the health professional team identify as important that should be followed up on, including resources for housing, interpretation, food and much more. And in the medical social sciences, a focus on language socialization can lead us to a more nuanced understanding of the clinical gaze and its production.

What might medical education draw from to counteract the exclusion of the social aspects of health and health care? Scholars and practitioners in *social medicine*, the interdisciplinary field that links the social sciences and medicine, recently published patient cases developed to teach the importance of social issues in the production, experience, and treatment of sickness (Social Medicine Consortium, n.d.; Stonington et al., 2018). We hope social medicine cases will not become just another exception to the rule, but rather influence the broader ways in which medicine conceptualizes and interacts with patients as people in social worlds, avoiding inadvertent misdiagnosis, mistreatment and harm (Holmes et al., 2020). Social medicine seeks to teach clinicians to develop not just a “differential” of the possible diagnoses, but also a “structural differential” training the interdisciplinary medical team’s gaze also onto the social, economic and political structures that may need amelioration or intervention (Seymour et al., 2018).

Different frameworks in social medicine teach clinicians to focus on the processes producing social and health inequity (Breilh, 2021, 2023; Harvey et al., 2022, 2023; Waitzkin et al., 2001), critical intercultural ways in which health care should respond (Breilh, 2021; Harvey et al., 2023), and the “structural competence” that includes “structural humility” to learn from patients and communities in movements toward social and health equity (Hansen & Metzl, 2019; Neff et al., 2017, 2020; Metzl & Hansen, 2014; Piñones-Rivera et al., 2023). It is within this broadened frame—including both the affective intersubjective understanding of the patient from the medical humanities and the social structural context of patients and communities from social medicine—that empathy may be re-thought beyond a performance or perhaps a different framework for understanding care could be developed altogether. A fuller version of intersubjective clinical interaction that includes social analysis, then, may counteract burnout and moral injury while fostering solidarity with patients toward social and health equity.

## Footnotes

1 Names and titles of individuals have been changed.
